# Minimal One-Quarter Incision and Four-Step (MOQIF) Excision Method for Subcutaneous Lipoma

**DOI:** 10.3390/jcm15062448

**Published:** 2026-03-23

**Authors:** Seung Yun Oh, Seokchan Eun

**Affiliations:** 1 Faculty of Medicine, Dentistry and Health Sciences, The University of Melbourne, Parkville, Melbourne, VIC 3010, Australia; seungyuno@student.unimelb.edu.au; 2 Department of Plastic and Reconstructive Surgery, Seoul National University College of Medicine, Seoul National University Bundang Hospital, 82 Gumi-ro 173beon-gil, Bundang-gu, Seongnam 463-707, Republic of Korea

**Keywords:** lipoma, minimal incision, MOTIF, MOQIF, subcutaneous neoplasm, Vancouver Scar Scale

## Abstract

**Background:** Lipomas are common benign subcutaneous neoplasms treated surgically for cosmetic or symptomatic reasons. The minimal one-third incision and four-step (MOTIF) technique provides reliable excision with minimal scarring, but smaller proportional incisions remain unstudied. This study evaluates the minimal one-quarter incision and four-step (MOQIF) technique. **Methods:** Retrospective review of 82 patients undergoing MOQIF excision of histologically confirmed subcutaneous lipomas by a single surgeon from July 2024–December 2025 was done. Lipomas were stratified by maximum diameter: small-intermediate (<5 cm) and large (≥5 cm). MOQIF used a one-quarter incision of the lipoma’s long axis determined by preoperative ultrasound measurement and palpation with four steps: hydro dissection preserving superficial subcutaneous tissue, superficial dissection, staged deep dissection with selective cautery of fibrovascular septa, and intact mass delivery. Outcomes included excision length, postoperative complications, Vancouver Scar Scale (VSS) scores, recurrence, and subjective treatment satisfaction of patients. **Results:** Mean lipoma size was 6.8 ± 2.0 cm (75.6% ≥5 cm). All lipomas were completely excised through 1.69 ± 0.49 cm incisions (ratio 0.25). Complications were low: seroma 10.98% (16.7% vs. 9.4%, *p* = 0.404), hematoma 7.3% (11.1% vs. 6.3%, *p* = 0.608), with no infections, nerve injuries, or recurrences at a mean 8.9-month follow-up. VSS scores were equivalent between groups (0.83 vs. 1.06; *p* = 0.438) and overall patient satisfaction was high (3.54 ± 0.53 (2–4)). **Conclusions:** MOQIF achieves complete lipoma excision through one-quarter incisions with safety and cosmetic outcomes across lipoma sizes, demonstrating feasibility through standardized technique refinement and careful case selection.

## 1. Introduction

Lipomas represent the most common benign adipocytic neoplasm that frequently arises from the subcutaneous plane [1]. Although they may occur in deeper planes, only a small proportion extend into subfascial, intermuscular, or intramuscular compartments [2,3]. Regardless of depth, lipomas are typically benign and often remain asymptomatic for years [4]. Subcutaneous lipomas demonstrate characteristic anatomical distribution patterns, occurring most commonly in the trunk (35–40%) and extremities (30–35%), while facial lipomas remain relatively rare (2–5%) due to regional differences in adipocyte density and fascial architecture [1,3]. Indications for excision include esthetic distaste and symptoms related to mass effect on adjacent neurovascular structures, such as pain, paresthesia, discomfort, reduced muscle function, or impingement [2,5].

Surgical excision remains the gold standard of care due to its low recurrence rate and ability to provide a definitive histological diagnosis [6]. However, conventional open techniques may result in visible scarring that can be cosmetically unacceptable, especially in exposed areas of the body [7]. The central challenge in lipoma surgery is therefore to balance complete tumor removal with optimal cosmetic outcomes. While patients desire minimal scarring, reduced incision lengths can potentially increase the risk of complications and paradoxically produce hypertrophic scars [6]. Minimally invasive approaches present additional technical challenges, as smaller incisions inherently provide limited surgical visualization [8]. This reduced visualization is especially problematic given that lipomas are surrounded by a fibrous capsule and fibrovascular septa, which can predispose tumor fragmentation, residual tissue, and bleeding if not clearly visualized during dissection [9,10].

To address these competing priorities, multiple minimally invasive techniques have been described, including injection lipolysis, liposuction, and minimal scar extraction (MSE) [7,9,11]. Endoscopic approaches have also been explored, particularly for facial and forehead lipomas, and have demonstrated favorable cosmetic outcomes with hidden incisions [12]. Among these techniques, the minimal one-third incision and four-step (MOTIF) method proposed by Park [6] promises an algorithmic approach that achieves complete excision while minimizing scarring and complication rates. MOTIF utilizes an incision spanning one-third of the lipoma’s long-axis and excision is systematically done in four steps. The four sequential steps consist of tumescent infiltration, superficial circumferential dissection, deep circumferential dissection leaving a narrow stalk, and intact mass delivery.

Building upon this foundation, this study proposes a novel modification, the minimal one-quarter incision and four-step (MOQIF) technique, specifically designed for subcutaneous lipomas. MOQIF reduces the total incision length to one-quarter of the long axis while preserving the MOTIF four-step dissection sequence. As such, this new technique aims to further minimize incision length and visible scarring without compromising safety, complete excision, or overall clinical outcomes.

## 2. Materials and Methods

This retrospective chart review included patients who underwent surgical excision of histologically confirmed lipomas using the minimal one-quarter incision and four-step (MOQIF) technique at a single institution. From July 2024 to December 2025, a total of 82 patients underwent MOQIF. All procedures were performed under monitored anesthesia care (MAC) and performed by a single surgeon (S.C. Eun). The surgeon selected MOQIF for subcutaneous lipomas meeting specific clinical criteria: (1) size ≥2 cm on ultrasound measurement to justify surgical intervention; (2) well-defined subcutaneous plane on palpation and ultrasound without intramuscular extension; and (3) sufficient overlying skin laxity to permit delivery through reduced incisions. Cases with rigid overlying skin, suspicion of deep fascial involvement, or fascial locations requiring hidden-incision approaches were considered for alternative techniques. Patients with recurrent lipomas, suspected malignant soft-tissue tumors, or incomplete clinical records were excluded. Preoperatively, all patients underwent clinical examination and 7.5–12 MHz high-resolution linear ultrasound probe (GE 12L-RS, GE HealthCare, Chicago, IL, USA) to evaluate tumor size, depth, capsular integrity, and relationship to underlying fascia. Lesions demonstrating heterogenous echotexture, irregular margins, or deep muscular infiltration were referred for MRI evaluation to exclude liposarcoma before surgical planning. No preoperative biopsies were performed, as clinical and imaging characteristics were sufficient for surgical decision-making. Lipomas were stratified by maximum diameters into two size groups: small-intermediate (<5 cm) and large (≥5 cm). Data collected included patient demographics, tumor size and location, anesthesia type, intraoperative details, and postoperative complications (i.e., seroma, hematoma, nerve injury, and recurrence), scar quality, and follow-up duration.

### 2.1. MOQIF Technique

#### 2.1.1. Step I

**Figure 1:**
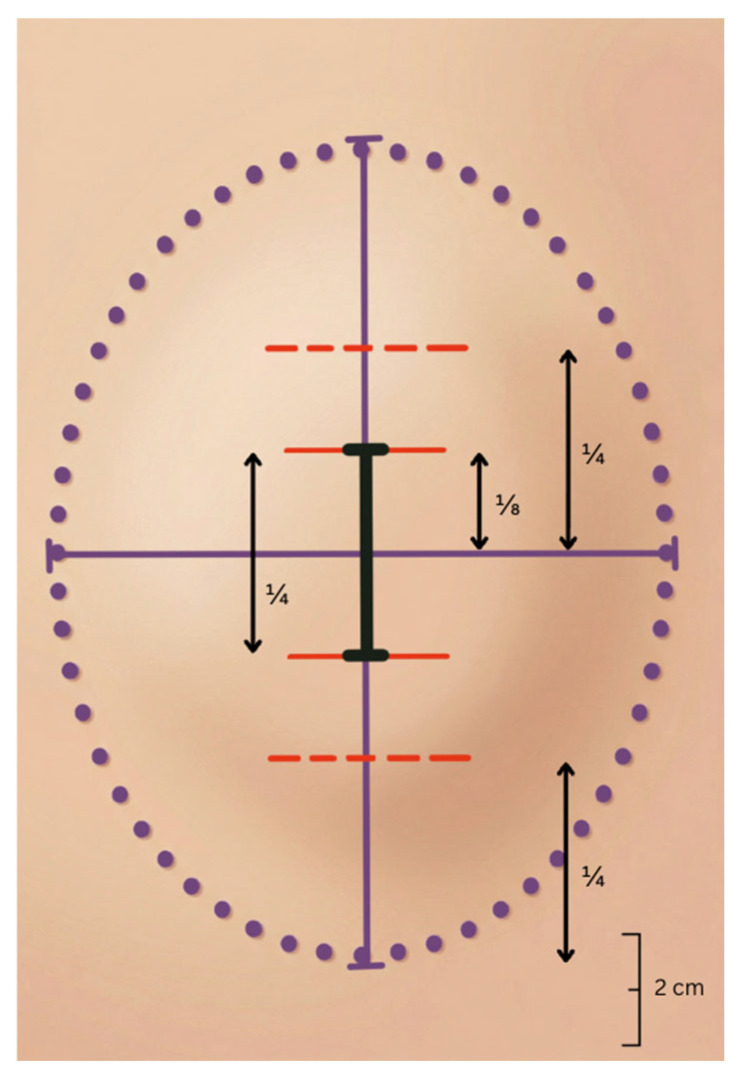
Orthogonal axis planning for MOQIF. The solid purple lines indicate the longitudinal and transverse axes of the lipoma; dotted red lines mark the one-fourth points, solid red lines the one-eighth points; the black line shows the planned incision measuring one-fourth of the maximal diameter of the lipoma. Scale bar = 2 cm.

The boundary of the lipoma was delineated using ultrasound measurement of the maximal diameter taken preoperatively combined with careful palpation. Two orthogonal axes (x and y) were drawn through the center of the mass to identify the long axis. The maximal diameter was divided into eight equal segments, and central skin incision corresponding to one-quarter of the diameter was planned between the one-eighth points on either side of the center (Figure 1). The incision line was systematically aligned with relaxed skin tension lines (RSTL) to optimize scar camouflage and minimize tension during wound closure [13].

Local anesthesia with 1% lidocaine and epinephrine (1:100,000) was administered generously depending on tumor size and patient factors to achieve hydro dissection around both superficial and deep surfaces of the lipoma. Following the principles of the original MOTIF method, at least five minutes were allowed for optimal vasoconstriction to minimize bleeding, allowing easier dissection of the mass [6]. Additional diluted solution with normal saline could be injected beneath the mass for larger lipomas to facilitate dissection and reduce systemic anesthetic and vasoconstrictor load.

A skin incision was made along the pre-marked one-fourth length using a No. 15 scalpel blade, and dissection was carried through the subcutaneous tissue until the superficial surface of the lipoma was exposed.

#### 2.1.2. Step II

Using forceps (e.g., Adson or Adson-Brown forceps (Ethicon, Johnson & Johnson, New Brunswick, NJ, USA)) in the nondominant hand, blunt dissection of the superficial surface of the lipoma was performed in a circumferential manner using Metzenbaum scissors or mosquito forceps (Ethicon, Johnson & Johnson, New Brunswick, NJ, USA). Electrocautery was deliberately avoided at this stage to minimize thermal injury to the fibrous capsule and reduce seroma formation risk [14]. All fibrous adhesions between the fibrous capsule and the lipoma were released to allow maximal mobilization of the mass within the pocket. Conceptually, this step can be visualized as separating the “yolk” (lipoma) from the “white” (fibrous capsule) without disrupting the integrity of the yolk.

#### 2.1.3. Step III

**Figure 2:**
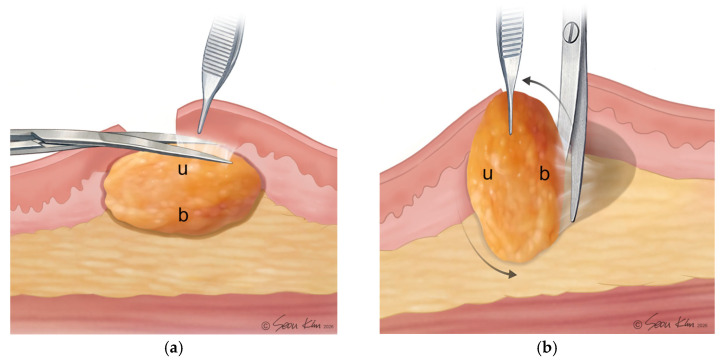
(**a**) Step II: Circumferential superficial dissection of the lipoma. Adson-Brown forceps are used to gently elevate the epidermal and dermal layers, while Metzenbaum scissors are employed for blunt dissection of the superficial fibrous adhesions (u). (**b**) Step III: Circumferential deep dissection of the lipoma. The mass is carefully mobilized using Adson-Brown forceps and Metzenbaum scissors to release deeper fibrous adhesions (b) prior to extraction.

Deep dissection was initiated at the inferior aspect of the short axis of the lipoma and continued as far as exposure allowed. Gentle rotation of the dominant wrist was used to obtain a more ergonomic and controlled approach to the deep surface. Dissection was then performed from the opposite end of the short axis to complete mobilization from below. Circumferential deep dissection continued along the axial margins until all fibrous adhesions between the capsule and the lipoma were freed, allowing the mass to “sink” into the cavity and facilitating subsequent extraction. During this step, fibrovascular septa were selectively divided using bipolar cautery to maintain hemostasis while limiting collateral thermal damage. Figure 2 illustrates the distinction between Step II and Step III, highlighting the superficial and deep planes of circumferential dissection.

#### 2.1.4. Step IV

After adequate superficial and deep mobilization, the lipoma was gently expressed and delivered through the central one-fourth incision. Progressive, controlled traction was applied to gradually dilate the skin opening while avoiding excessive force that could cause wound margin compression or ischemia. Self-retaining retractors were used minimally, and skin edges were protected with moist gauze during extraction to prevent mechanical injury. As the mass was delivered, bipolar cautery was used as needed to divide any remaining deep fibrovascular septa and achieve complete release. The resulting cavity was irrigated and meticulous hemostasis was confirmed. A silastic drain was placed for lipomas larger than 3 cm or for lesions located on the trunk and extremities, where dead space was more substantial. Wounds were closed in layered fashion using 3-0 or 4-0 absorbable sutures for deep dermal approximation to eliminate dead space, followed by 5-0 monofilament sutures for skin closure to optimize cosmetic outcomes. External dressings were applied to minimize shear forces and support optimal wound healing.

### 2.2. Postoperative Complications and Vancouver Scar Scale Analysis

Postoperative complications were recorded, including seroma, hematoma, infection, nerve injury assessed by clinical sensorimotor examination at each follow-up visit, and clinical recurrence during follow-up. Scar quality was assessed at a minimum of six months postoperatively using the Vancouver Scar Scale (VSS), in line with the methodology of the original MOTIF study, by a single evaluator to reduce interobserver variability. Independent observer scar assessment was not performed in this initial case series due to resource constraints and the retrospective study design. Incision length relative to tumor size, total follow-up time (in months), and overall patient satisfaction were also documented to evaluate whether the minimal one-quarter incision approach conferred additional cosmetic or functional benefits compared with previously reported one-third incisions.

### 2.3. Postoperative Scar Management

All patients received standardized postoperative scar management beginning at 2 weeks post-operation once wound healing was confirmed. The protocol included: (1) silicone gel sheet application for 12–23 h daily for 3–6 months; (2) paper tape reinforcement along the incision line to reduce tension during the first 3 months; (3) daily sun protection using SPF 50+ sunscreen during the healing phase; and (4) gentle scar massage after 4 weeks to promote collagen remodeling. Patients were instructed to avoid heavy lifting and strenuous activity involving the surgical site for 4 weeks to minimize tension on the healing incision.

### 2.4. Overall Treatment Outcome

Treatment outcomes were assessed based on subjective patient-reported satisfaction, encompassing postoperative recovery, functional outcome, and esthetic appearance. Outcomes were graded using a four-point ordinal scale: poor, satisfactory, good, excellent. For descriptive analysis, these categories were assigned numerical scores from 1 (poor) to 4 (excellent).

### 2.5. Statistical Analysis

All statistical analyses were performed using standard statistical software (e.g., RStudio 3.6.0+ and Microsoft Excel). Continuous variables such as patient age, lipoma size, incision length, follow-up duration, and Vancouver Scar Scale (VSS) scores were summarized as means with standard deviations and ranges. Categorical variables, including sex, lipoma location, size group, post-operative complications (seroma, hematoma, infection, nerve injury, recurrence), and treatment outcome were presented as frequencies and percentages.

To compare outcomes among the two lipoma size groups (<5 cm and ≥5 cm), independent two sample t-tests (Welch’s) were used for continuous variables after checking for normality, and chi-square or Fisher’s exact tests were applied to categorical variables.

For binary outcomes, complication rates were reported with 95% confidence intervals calculated using the Wilson score method. For continuous outcomes, mean differences and 95% confidence interval were obtained from Welch’s two-sample t-test. All statistical tests were two-tailed, and a *p*-value of less than 0.05 was considered statistically significant.

## 3. Results

### 3.1. Patient Demographic and Lesion Characteristics

A total of 82 patients underwent subcutaneous lipoma excision using the MOQIF method. The mean patient age was 55.2 ± 12.4 years. The cohort included both male and female patients, with majority of lipomas located at the trunk (*n* = 48; 58.5%) and extremities (*n* = 32; 39.0%), followed by the face (*n* = 2; 2.4%).

**Figure 3:**
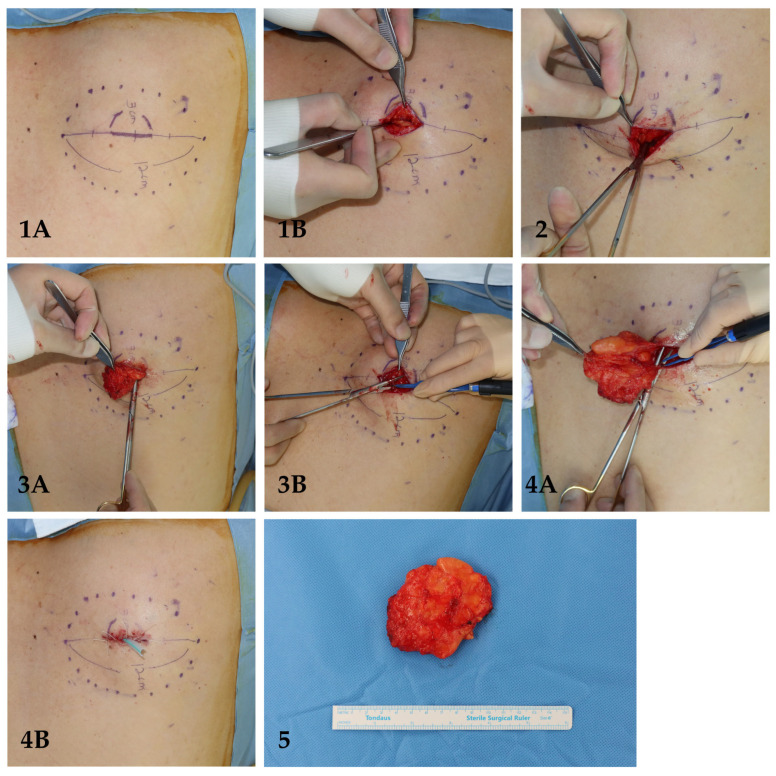
Case 1: Subcutaneous lipoma located at the right posterior shoulder. (**1A**) Step I: lipoma boundary made after deep palpation of borders; (**1B**) step I: incision to reveal superficial surface of lipoma; (**2**) step 2: circumferential superficial dissection of lipoma; (**3A**) step 3: circumferential deep dissection of lipoma; (**3B**) step 3: electrocautery to release fibrovascular septa of lipoma; (**4A**) step IV: delivery of lipoma and separation of deep fibrovascular septa using electrocautery; (**4B**) step IV: silastic drain insertion; (**5**) excised lipoma.

**Figure 4:**
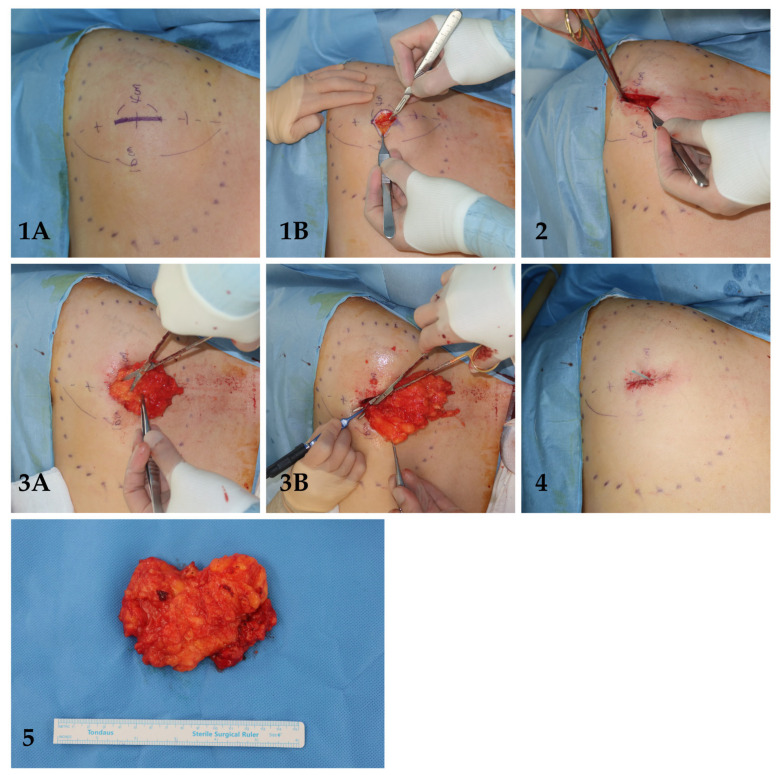
Case 2: Subcutaneous lipoma located at the left posterior shoulder. (**1A**) Step I: lipoma boundary made after deep palpation of borders; (**1B**) step I: incision to reveal superficial surface of lipoma; (**2**) step 2: circumferential superficial dissection of lipoma; (**3A**) step 3: circumferential deep dissection of lipoma; (**3B**) step 3: electrocautery to release fibrovascular septa of lipoma; (**4**) step IV: silastic drain insertion; (**5**) excised lipoma.

The mean lipoma size was 6.8 cm, and when further stratified 22.0% were in the small-intermediate group (<5 cm) and 78.0% were in the large group (≥5 cm). The mean incision length was 1.69 ± 0.49 cm, corresponding to an average incision-to-tumor size ratio of 0.25. All lipomas were successfully excised without the need for incision extension (Table 1). Figure 3 and Figure 4 illustrate a step-by-step of two cases of lipoma excision utilizing the MOQIF method.

### 3.2. Post-Operative Complications and Vancouver Scar Scale Analysis

Overall complication rates were low, with only 9 seroma (10.98%) and 6 hematoma (7.3%) formations in the cohort. There were no cases of infections, nerve injuries, or recurrences during the follow-up period. No cases of wound edge ischemia, delayed healing, or hypertrophic scarring related to mechanical traction during lipoma delivery were observed in this series. Furthermore, all complications were managed conservatively without any long-term sequelae.

When stratified by size group, seroma formation occurred in 16.7% of patients in the small–intermediate group (95% CI = 5.8–39.2%) and 9.4% in the large group (95% CI = 4.4–19.0%), with no statistically significant difference between groups (*p* = 0.404). Hematoma formation occurred in 11.1% of the small-intermediate group (95% CI = 3.1–33.0%) and 6.3% in the large group (95% CI = 2.5–15.0%), with no statistically significant difference (*p* = 0.608) (Table 2).

### 3.3. Scar Assessment, Follow-Up, and Overall Treatment Outcome

**Figure 5:**
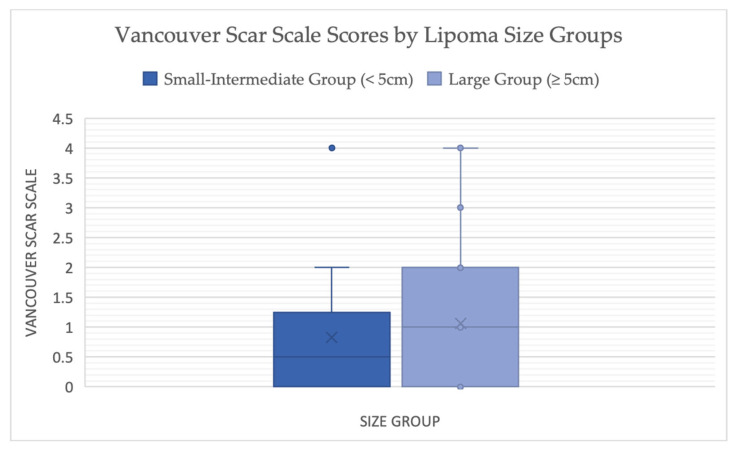
Comparison using Box-and-whisker plot of Vancouver Scar Scale scores between Two Size groups. (Welch’s Two Sample Independent t-tests, *p* = 0.438; 95% CI = −0.83–0.37).

Scar assessment, using VSS scores, was favorable in both size groups, indicating favorable scar quality and outcomes. The mean VSS was 0.83 ± 1.10 in the small–intermediate group and 1.06 ± 1.07 in the large group. The mean difference (small–intermediate–large) was −0.23 (95% CI = −0.83–0.37; *p* = 0.438), indicating no statistically significant difference (Figure 5).

The mean follow-up duration for the cohort was 8.9 months, with no lipoma recurrences. Subjective treatment satisfaction was high across the cohort. Global treatment outcome based on recovery, functional outcome, and esthetic appearance was 3.54 ± 0.53 (2–4), corresponding to outcomes rated as good to excellent. No patients reported poor outcomes, and majority of patients rated their overall treatment outcome as excellent.

## 4. Discussion

The surgical management of subcutaneous lipomas has evolved significantly with the development of minimal incision techniques that prioritize both complete tumor removal and optimal cosmetic outcomes. The minimal one-third incision and four-step method (MOTIF), as described by Park [6], established a benchmark for minimal incision lipoma surgery, demonstrating 100% complete excision rates across all lipoma sizes with consistent low complication rates and scar quality regardless of tumor dimensions. However, despite the success of the original MOTIF technique, a critical gap exists in the literature regarding even more conservative incision approaches.

Other minimally invasive strategies, such as liposuction and lipolysis injection, offer minimal scar formation due to the utilization of tiny access ports, but they provide virtually no direct visualization of the tumor. This increases the risk of lipoma fragmentation, incomplete excision, and potential recurrence, especially when malignancy cannot be excluded preoperatively [15]. Endoscopic approaches similarly achieve minimal scarring and have demonstrated very low recurrence rates, but they are associated with longer operative times, higher procedural costs, and a need for specialized equipment and training, limiting their availability to highly trained subspecialty surgeons [12]. Against this background, a simple open technique that preserves direct visualization while further reducing incision length, such as the MOQIF modification of MOTIF, may offer a more broadly applicable balance between safety, efficiency, and cosmetic benefit.

Notably, Sakamoto [16] demonstrated successful complete resection of large lipomas (5–12 cm) using fixed 1-inch incisions, representing proportional incisions (approximately 21% of tumor diameter), confirming that proportional incisions well below the traditional one-third can achieve complete excision. Similarly, Kang [17] analyzed 122 lipomas and found that anatomical location, rather than tumor size and depth, was the primary determinant of minimal achievable incision length during extraction, supporting the feasibility of further incision reduction for trunk and extremity lesions. These findings provide a strong rationale for the present MOQIF technique, which standardizes a one-quarter incision specifically for subcutaneous lipomas in these common sites.

The present study demonstrates that MOQIF achieves reliable outcomes in well-selected subcutaneous lipomas. Complete excision was accomplished in all 82 cases through mean incisions of 1.69 cm, yielding an incision-to-tumor ratio of 0.25. The higher mean lipoma size (6.8 cm) reflects the surgeon’s case selection strategy during this initial case series, where MOQIF was preferentially applied to larger lesions after gaining confidence with the technique. Smaller lipomas (<2 cm) were often managed with observations or alternative approaches, as the proportional benefit of minimal incision techniques diminishes for very small lesions. Complication rates remained size-independent, with seroma and hematoma managed conservatively without sequelae. No infections, nerve injuries, or recurrences were observed during mean 8.9-month follow-up. Vancouver Scar Scale scores showed no significant differences between size groups, suggesting that proportional reduction to one-quarter did not compromise scar outcomes. These results indicate that further incision reduction from one-third (MOTIF) to one-quarter (MOQIF) is feasible without increasing morbidity when applied to appropriate subcutaneous lesions.

However, these findings should be interpreted within the context of this study’s methodology. This was a retrospective single-surgeon case series without a direct MOTIF control group, limiting definitive comparative conclusions regarding superiority of one-quarter versus one-third incisions. The observed outcomes reflect both the technical refinement of MOQIF and the surgeon’s deliberate case selection during this initial experience. The favorable results may therefore represent combined effects of technique optimization and patient selection rather than technique alone. Prospective comparative trials with randomization to MOQIF versus MOTIF would be required to isolate the independent effect of incision length on clinical outcomes.

In accordance with the established literature, the majority of lipomas in this series were located on the trunk (58.5%) and extremities (39.0%), whereas facial lipomas were relatively rare (2.4%) [1,3]. Although the two facial cases in this series were successfully managed with the MOQIF technique and had met the inclusion criteria of being well-encapsulated, ≥2 cm, and presenting with sufficient skin laxity, their limited representation in this series precludes definitive conclusion regarding the technique’s broader applicability to facial lipomas. Consequently, the primary utility of MOQIF shall remain focused on trunk and extremity lesions, where skin redundancy in these regions facilitates the delivery of large tumors through minimal incisions [17]. Furthermore, given the heightened esthetic and anatomical constraints associated with the face, endoscopic or other hidden incision approaches may remain preferrable in such cases, provided the necessary expertise and resources are available [12].

The applicability of MOQIF across different lipoma subtypes warrants careful consideration. This technique was developed primarily for well-encapsulated subcutaneous lipomas with distinct fibrous capsules that permit clean circumferential dissection. In poorly encapsulated or non-encapsulated lipomas, the absence of a clear dissection plane increases the risk of fragmentation and incomplete excision through minimal incisions, potentially compromising oncologic adequacy [18]. Similarly, multisegmented or lobulated lipomas present greater technical difficulty due to irregular architecture and multiple fibrovascular attachments, which may require larger incisions for safe visualization and complete removal [8,19]. In such cases, selective modification of the incision length or consideration of alternative approaches may be warranted to prevent tumor fragmentation and ensure complete excision.

MOQIF is specifically designed for subcutaneous lipomas and should not be extended to intramuscular lipomas. Intramuscular lesions require deeper dissection with risk of neurovascular injury, greater hemorrhage potential, and more challenging margin control due to infiltration into muscle fibers [20,21]. The limited visualization inherent to minimal incision techniques is incompatible with the technical demands of intramuscular lipoma resection, where wide exposure is necessary for safe identification and preservation of critical structures. Intramuscular lipomas therefore remain an absolute contraindication of MOQIF and should be managed through conventional open approaches with adequate exposure.

When selecting between MOTIF and MOQIF, several clinical factors should guide decision making. MOQIF is appropriate for: (1) well-encapsulated subcutaneous lipomas ≥2 cm confirmed by ultrasound; (2) trunk or extremity locations with sufficient skin laxity; (3) mobile overlying skin that permits tissue expansion during extraction; (4) absence of concerning features for malignancy or deep invasion. Conversely, MOTIF (one-third incision) may be preferable for: (1) lipomas in areas with limited skin laxity (e.g., anterior leg, dorsal hand); (2) poorly encapsulated or lobulated lipomas requiring greater visualization; (3) lipomas in proximity to neurovascular structures where precise dissection is critical; and (4) first-time minimal incision operators building technical proficiency. These criteria provide a practical framework for technique selection based on anatomic and technical considerations.

Several technical considerations are critical to the success of the MOQIF method. During Step I, preoperative ultrasound measurement combined with meticulous palpation and marking using orthogonal axes standardizes the one-quarter incision design and helps ensure the centralization of the incision. Systematic attention to RSTL alignment, combined with layered wound closure technique and standardized postoperative scar management (silicone gel, paper tape, sun protection, and gel massage), collectively optimize final scar quality beyond the contribution of incision length alone. Similar to the original MOTIF paper, generous tumescent infiltration and adequate waiting time allow effective hydro dissection, reduce bleeding, and facilitate atraumatic dissection [6]. Furthermore, during dissection through the subcutaneous tissue preservation of the superficial subcutaneous layer must be deliberately done to prevent postoperative skin indentation or contour depression. This is a recognized complication of aggressive tissue undermining in minimal incision surgery [16].

In Step II, mobilization of the mass with forceps grasping the fibrous capsule, rather than retractors or direct manipulation of the fatty lobules, improves visualization of the circumferential pocket and reduces fragmentation of the lipoma.

In Step III, staged deep dissection from both ends of the short axis allows the surgeon to work within a constrained field more ergonomically and promotes symmetric mobilization of the mass. Selective use of bipolar cautery to divide fibrovascular septa maintains hemostasis while limiting thermal injury to surrounding tissues, which is particularly important given the association between excessive cautery and seroma formation [14].

In Step IV, controlled progressive traction during lipoma delivery is essential to prevent wound margin complications. Excessive or abrupt force can cause compression injury to wound edges, resulting in tissue ischemia, delayed healing, or hypertrophic scarring [22,23]. In this series, deliberate attention to gentle, staged extraction with skin edge protection prevented such complications, as evidenced by the absence of wound healing issues or inferior scar quality. This mechanical consideration underscores the importance of surgical technique refinement beyond incision size alone.

This study has several limitations. Its retrospective, single-center design and absence of a direct MOTIF control group restrict definitive comparative conclusions. The decision to use MOQIF was made by a single experienced surgeon based on intraoperative assessment of skin laxity and tumor characteristics, introducing potential selection bias. The favorable outcomes observed may therefore reflect both the technical merits of MOQIF and deliberate case selection for subcutaneous lipomas most amendable to minimal incision extraction. A prospective randomized trial comparing MOQIF to MOTIF with standardized inclusion criteria would be necessary to isolate the independent effect of incision length reduction and establish superiority or equivalence with greater confidence.

The follow-up period (mean 8.9 months) was sufficient to capture immediate postoperative complications and assess scar maturation, but may be inadequate to detect late recurrences, particularly for incompletely excised lesions or atypical lipomatous tumors. Lipomas generally have low recurrence rates when completely excised [7,11] but longer-term surveillance (≥2–3 years) would strengthen conclusions regarding oncologic adequacy. Additionally, the absence of independent observer scar assessment represents a methodological limitation that may introduce bias, as single-evaluator VSS scoring lacks the objectivity of blinded third-party assessment. Future prospective studies should incorporate standardized photography protocols and independent scar evaluation to provide robust cosmetic outcome data.

The reproducibility of MOQIF beyond the original surgeon remains unestablished. All procedures were performed by a single surgeon experienced in minimal-incision lipoma excision, which may limit generalizability to less experienced operators. The consistent outcomes observed across 82 consecutive cases using a standardized four-step protocol suggest technical reproducibility is achievable, but this hypothesis requires validation through multi-surgeon, multi-center studies. Training protocols and learning curve analyses would be valuable to establish the educational requirements for safe adoption of MOQIF by surgeons with varying levels of minimal-incision surgery experience.

Long-term photographic documentation would significantly enhance the evidential basis for cosmetic claims. While VSS scoring provides quantitative scar assessment, standardized long-term photographs (≥6–12 months postoperatively) would allow readers to independently evaluate scar outcomes and assess the esthetic benefits of one-quarter incisions compared to conventional approaches. Future publications should include representative long-term images demonstrating scar appearances across different anatomical locations and lipoma sizes to facilitate broader evaluation of the technique’s cosmetic advantages.

Nonetheless, the present findings provide preliminary evidence that a standardized one-quarter incision, four-step approach can safely extend minimal-incision principles while preserving direct visualization, and they support further prospective and comparative studies to refine indications and validate long-term outcomes for the MOQIF technique.

## 5. Conclusions

In conclusion, the MOQIF technique represents a safe and potentially reproducible refinement of minimally invasive lipoma excision for well-selected subcutaneous lipomas. By achieving complete tumor removal through a one-quarter incision without increasing complication rates in this initial case, this method offers a valuable alternative for surgeons seeking to further minimize surgical footprint while preserving excellent clinical outcomes when applied to appropriate trunk and extremity lesions. Further prospective comparative studies with standardized patient selection, independent scar evaluation, and longer follow-up are warranted to validate these preliminary findings and establish the broader applicability and reproducibility of MOQIF across diverse surgical settings.

## Figures and Tables

**Table 1:** Patient demographic and treatment outcome.

Patient Demographics Variables	Values
No. of Patients	82
Mean Age ± SD (range), years	55.2 ± 12.4 (33–86)
Sex	
Male	52 (63.4%)
Female	30 (36.5%)
Location	
Trunk	48 (58.5%)
Extremities	32 (39.0%)
Face	2 (2.4%)
Mean Size ± SD (range), cm	6.8 ± 2.0 (2.2–9.9)
Mean Incision Size ± SD (range), cm	1.69 ± 0.49 (0.55–2.45)
Size	
<5 cm	18 (22.0%)
≥5 cm	64 (78.0%)
Mean Follow-up ± SD (range), months	8.9 ± 3.9 (3–18)
Overall Treatment Outcome	3.54 ± 0.53 (2–4)

**Table 2:** Postoperative complications using Fischer’s exact test.

Complication	<5 cm (*n* = 18)	≥5 cm (*n* = 64)	Total (*n* = 82)	*p*-Value
Seroma	3 (16.7%)	6 (9.4%)	9 (10.98%)	0.404
Hematoma	2 (11.1%)	4 (6.3%)	6 (7.3%)	0.608
Nerve Injury	0	0	0	-
Recurrence	0	0	0	-

## Data Availability

The data collected and analyzed can be requested from the corresponding author on reasonable request.

## References

[1] Kim K.-H., Kwon S.-H., Sim W.-Y., Lew B.-L. (2021). The Study of Relationship between Anatomical Sites and Depth of the Lipoma. Ann. Dermatol..

[2] Snow E.L., Nelson S., White A.C. (2023). Muscular, Vascular, and Neurological Impacts from a Giant Lipoma in the Arm. Transl. Res. Anat..

[3] Rydholm A., Berg N.O. (1983). Size, Site and Clinical Incidence of Lipoma: Factors in the Differential Diagnosis of Lipoma and Sarcoma. Acta Orthop. Scand..

[4] Rauh J., Klein A., Baur-Melnyk A., Knösel T., Lindner L., Roeder F., Jansson V., Dürr H.R. (2018). The Role of Surgical Margins in Atypical Lipomatous Tumours of the Extremities. BMC Musculoskelet. Disord..

[5] Stebbins W.G., Hanke C.W., Petersen J. (2011). Novel Method of Minimally Invasive Removal of Large Lipoma after Laser Lipolysis with 980 Nm Diode Laser: Lipoma Removal Using 980 Nm Diode Laser. Dermatol. Ther..

[6] Park J.K., Kim J., Kim J.-H., Eun S. (2021). Minimal One-Third Incision and Four-Step (MOTIF) Excision Method for Lipoma. BioMed Res. Int..

[7] Chandawarkar R.Y., Rodriguez P., Roussalis J., Tantri M.D. (2005). Minimal-Scar Segmental Extraction of Lipomas: Study of 122 Consecutive Procedures. Dermatol. Surg..

[8] Won J.H., Hur K., Ohn J., Mun J. (2020). Surgical Management of Lipomas: Proposal of the Z-incision Design and Surgical Algorithm Based on Tumor Size. Dermatol. Ther..

[9] Al-basti H.A., El-Khatib H.A. (2002). The Use of Suction-Assisted Surgical Extraction of Moderate and Large Lipomas: Long-Term Follow-Up. Aesthetic Plast. Surg..

[10] Vagholkar K., Vagholkar S., Purandare T. (2024). Lipoma: Neoplasm beyond Boundaries. Int. Surg. J..

[11] Copeland-Halperin L.R., Pimpinella V., Copeland M. (2015). Combined Liposuction and Excision of Lipomas: Long-Term Evaluation of a Large Sample of Patients. Plast. Surg. Int..

[12] Sun Y., Wang Q., Xie H. (2025). Minimally Invasive Removal of Frontal Lipoma with Endoscope. Aesthetic Plast. Surg..

[13] Borges A.F. (1984). Relaxed Skin Tension Lines (RSTL) versus Other Skin Lines. Plast. Reconstr. Surg..

[14] Keogh G.W., Doughty J.C., McArdle C.S.M., Cooke T.G. (1998). Seroma Formation Related to Electrocautery in Breast Surgery: A Prospective Randomized Trial. Breast.

[15] Malisetyan T., Harmon S., Zhong N., Tatarian G. (2024). Familial Multiple Lipomatosis: A Case Report. Am. J. Cosmet. Surg..

[16] Sakamoto A., Okamoto T., Matsuda S. (2018). Subcutaneous Lipomas: A Minimally Invasive Method for Resection of Subcutaneous Lipomas Preserving Retaining Ligaments. Eur. J. Plast. Surg..

[17] Kang D.-H., Lew B.-L., Kwon S.-H. (2023). Do the Clinical Characteristics of Lipomas Influence the Incision Length During Minimal Incision Extraction?. J. Cutan. Med. Surg..

[18] Do W.H., Choi Y.W. (2019). Complications of the Surgical Excision of Encapsulated versus Nonencapsulated Lipomas: A Retrospective Analysis. Arch. Aesthetic Plast. Surg..

[19] Mehrotra S., Bhatia M., Rana V. (2015). Giant Recurrent Lipoma of Trunk Weighing Eight Kilograms. Med. J. Armed Forces India.

[20] Akiyama G., Ono S., Sekine T., Usami S., Ogawa R. (2022). A Scoring System That Predicts Difficult Lipoma Resection: Logistic Regression and Tenfold Cross-Validation Analysis. Dermatol. Ther..

[21] McTighe S., Chernev I. (2014). Intramuscular Lipoma: A Review of the Literature. Orthop. Rev..

[22] Ogawa R., Téot L., Mustoe T.A., Middelkoop E., Gauglitz G.G. (2020). Mechanobiology of Cutaneous Scarring. Textbook on Scar Management: State of the Art Management and Emerging Technologies.

[23] Han H.S., Choi S.Y., Kim W.-S., Choi Y.-J., Yoo K.H. (2023). A Narrative Review of Scar Formation. Med. Lasers.

